# Prophylactic HPV vaccination in HPV‐related gynecologic cancers: European Society of Gynecological Oncology (ESGO) prevention committee opinion

**DOI:** 10.1002/ijgo.16120

**Published:** 2024-12-30

**Authors:** Nicolò Bizzarri, Maria Kyrgiou, Rosa De Vincenzo, Ignacio Zapardiel, Zoia Razumova, Nadja Taumberger, Ico Toth, Charalampos Theofanakis, Murat Gultekin, Elmar A. Joura

**Affiliations:** ^1^ UOC Ginecologia Oncologica, Dipartimento di Scienze Della Salute Della Donna, del Bambino e di Sanità Pubblica Fondazione Policlinico Universitario Agostino Gemelli IRCCS Rome Italy; ^2^ Department of Metabolism, Digestion and Reproduction, Faculty of Medicine Imperial College London London UK; ^3^ West London Gynaecological Cancer Centre Imperial College Healthcare NHS Trust London UK; ^4^ Università Cattolica del Sacro Cuore Rome Italy; ^5^ Gynecologic Oncology La Paz University Hospital Madrid Spain; ^6^ Department of Women's and Children's Health Karolinska Institutet Stockholm Sweden; ^7^ Department of Obstetrics and Gynecology Medical University of Graz Graz Austria; ^8^ Hospital Spittal a d Drau Spittal a d Drau Carinthia Austria; ^9^ Mallow Flower Foundation Dunaharaszti Hungary; ^10^ Department of Gynecological Oncology General Hospital of Athens Alexandra Athens Greece; ^11^ Division of Gynecological Oncology, Department of Obstetrics and Gynecology Acettepe University Faculty of Medicine Ankara Türkiye; ^12^ Department of Gynecology and Obstetrics, Comprehensive Cancer Center Medical University of Vienna Austria

**Keywords:** cervical cancer, gynecologic cancer, HPV, vaccination, vaginal cancer, vulvar cancer

## Abstract

Many clinicians recommend that patients diagnosed with HPV‐related gynecologic cancers receive prophylactic HPV vaccination at the time of cancer diagnosis or after cancer treatment. In view of the large use of such practice, we aimed to assess the literature evidence supporting the use of prophylactic HPV vaccines after diagnosis or treatment of HPV‐related gynecologic cancers. Women who develop HPV‐related cervical, vaginal, and vulvar cancers represent a subgroup of patients who may be particularly sensitive to HPV infection and re‐acquire infections. The rationale that the use of prophylactic HPV vaccination at the time or after treatment for cervical, vaginal, and vulvar cancers might reduce the risk of future HPV‐related diseases might be explained by the data coming from the use of HPV vaccination after treatment of pre‐invasive disease; however, the evidence on the use of HPV vaccination in the setting of HPV‐related gynecologic cancers is currently absent. In this context, observational and experimental studies document an important drop in effectiveness of HPV vaccination by age. Physicians should be aware of catch‐up programs in their countries and should be ready to counsel patients about prophylactic HPV vaccine efficacy according to their age. In general, no evidence exists supporting the use of prophylactic HPV vaccine in patients diagnosed with HPV‐related gynecologic cancers; therefore, the European Society of Gynecological Oncology (ESGO) prevention committee opinion is to counsel these patients as any HPV‐related non‐gynecologic cancer (such as anal or oropharyngeal cancer) and non‐cancer patient, suggesting vaccination according to patient's age and prognosis, knowing there is a decrease of efficacy with increasing age. Studies on the use of prophylactic HPV vaccine in patients diagnosed with HPV‐related gynecologic cancers are strongly needed.

## Synopsis

No evidence supports the use of HPV vaccination in patients diagnosed with HPV‐related gynecologic cancers; they should be counseled according to age and prognosis.

## 1 INTRODUCTION

Vaccination against HPV before coital onset significantly reduces the incidence of invasive cancer and its precursors.^1^, ^2^, ^3^, ^4^, ^5^, ^6^ The European Society of Gynecological Oncology (ESGO) and other professional societies, such as the European Federation of Colposcopy (EFC) and the Italian Society of Colposcopy and Cervico‐Vaginal Pathology (SICPCV), support gender‐neutral vaccination programs for children and young adolescents, with a catch‐up program for older teenagers and young adults, if feasible, and vaccination in older women on an individual basis.^5^, ^7^, ^8^ This can be contextualized in a more general view of the World Health Organization (WHO), which released a recent position paper recommending that HPV vaccines should be included in all national immunization programs and should reach a coverage of 90% of all girls by the age of 15 by 2030. WHO stated that prevention of cervical cancer is best achieved through the immunization with HPV vaccines of girls and boys before they become sexually active, as they have excellent safety profiles, and they are highly efficacious.^9^ Recent population‐based linkage studies joining individual patient data from HPV vaccination and cancer registries confirm excellent protection against invasive cervical cancer and corroborate the findings from randomized trials that demonstrated excellent protection against HPV infection and associated cervical pre‐cancer. However, protection clearly decreases by age at vaccination and HPV exposure.^10^ With this opinion paper, we aimed to provide an overview of the current evidence on the use of prophylactic HPV vaccines after diagnosis and treatment of HPV‐related gynecologic cancers.

## 2 GENERAL CONSIDERATIONS FOR CANCER PATIENTS

In patients with a cancer diagnosis and treatment, the focus is usually set on treatment and follow up of their cancer, so prevention of other medical conditions is sometimes neglected.

Patients are at higher risk for a second malignancy after a cancer treatment, especially after irradiation and chemotherapies.^11^ This is a vulnerable population that probably needs more protection against infections than the general population, and at least the recommendations for general populations should be obeyed. A recent survey aimed to assess the implementation barriers of providing the HPV vaccine in cancer patients: the 24 interviewed oncology providers identified educational barriers to HPV vaccination and complicated post‐treatment HPV vaccination guidelines as the main limitation for HPV vaccination in cancer patients,^12^ thus showing the lack of evidence about use of HPV vaccination in cancer patients.

Moreover, in pediatric, adolescent, and young adult (defined as individuals <40 years of age) cancer survivors the risks of secondary cancers associated with HPV infection are as much as three times higher compared with the general population, because of the long‐lasting immunosuppressive effects of cancer therapy (i.e. chemotherapy, radiation, bone marrow transplantation, and immunotherapy). Despite this, HPV vaccination rates in this group of patients remain low with consequent interest in potential strategies to increase the vaccine coverage.^13^ HPV vaccination in patients with previous HPV‐related cancer might confer immune stimulation with prevention of other sites of infection/elimination of colonizing HPV. Nevertheless, evidence in support of this is lacking.

General life expectancy is increasing and in cancer survivors with a good prognosis, there are at least a couple of decades ahead during which these patients should be protected. One should keep in mind that the HPV vaccines have the potential to prevent six different cancers (cervical, vaginal, vulvar, anal, penile, and oropharyngeal cancer) and their precursors in the genital tract (transmission by sexual contact) such as: high‐grade squamous intraepithelial lesions (HSIL) of the cervix, vulva, vagina and anus, as well as anogenital warts. In countries with cervical cancer screening in place (either HPV testing or cytology) the vaccine prevents incidence of disease and reduces the stress and fear of the individual and the subsequent cost for the public. HPV vaccination reduces the burden of HPV infection, associated disease, cost of follow‐up and treatment. In Europe, there is no upper age limit for the use of HPV vaccines, but there are age restrictions in reimbursement. It should be highlighted that the efficacy of prophylactic HPV vaccine is the highest in individuals vaccinated at 12–13 years of age with an increased cumulative incidence of cervical cancer in those vaccinated after 14 years of age.^10^ Interestingly, recent evidence showed that HPV vaccination can be started at an even younger age (9 years), with a study concluding that proactive initiation of vaccination at 9–10 years of age was associated with higher rates of series completion by 13 years of age.^14^

## 3 PATIENTS WITH HPV‐RELATED DISEASE

Patients with HPV‐related disease have a long‐lasting increased risk (up to 20 years) for other subsequent HPV‐related diseases and HPV‐related cancer.^15^, ^16^, ^17^ Table 1 shows the studies reporting on outcomes from HPV infection after previous cancer diagnosis and treatment.^18^, ^19^, ^20^, ^21^, ^22^, ^23^ Recently, there has been growing interest in the use of prophylactic HPV vaccines after treatment of HPV‐related pre‐neoplastic diseases in order to prevent recurrences. In fact, the use of HPV vaccines after HPV exposure might prevent the patient from contracting the same or different types of HPV infection.^24^, ^25^ HPV vaccine is highly immunogenic, with the majority of the recipients achieving serum antibody titers that were approximately 40‐fold higher than those observed in natural infection.^26^ HPV vaccine might give protection against other HPV‐related diseases, give protection against re‐infection or re‐activation for seropositive people with a previously controlled infection, and give cross immunization toward other HPV genotypes.^15^, ^27^, ^28^, ^29^ Moreover, the surgical treatment of HPV‐related diseases might recover a local HPV‐naïve microenvironment (because of HPV clearance induced by treatment) where the vaccine could be effective.^30^ In this environment, HPV vaccines might stimulate cell‐mediated immunity, which may also play a role in preventing recurrent infection and might prevent auto‐inoculation across new exposed anatomic sites.^31^, ^32^ However, potential HPV clearance by the host‐immune reaction without the assistance of the HPV vaccine must also be considered, particularly in young individuals.

**TABLE 1:** Studies on outcomes from HPV infection after previous cancer diagnosis and treatment.

Author	Year	Journal	Setting	Type of article	Main objective	Number of patients	Main results	Main conclusion
Ojha et al.^18^	2013	*PLoS One*	Long‐term survivors of PAYA cancers	Retrospective (SEER database)	To describe the burden of HPV‐associated malignancies among survivors of PAYA	64 547	Compared with females in the general population, female PAYA cancer survivors had a 40% relative excess of HPV‐associated malignancies overall (standard incidence ratio, 1.4; 95% CI 1.2–1.8)	Increased HPV‐associated malignancies among PAYA cancer survivors compared with the general population. A portion of subsequent malignancies among PAYA cancer survivors may be directly attributable to HPV infection.
Suk et al.^19^	2018	*JAMA Netw Open*	Second primary cancers associated with HPV	Retrospective (SEER database)	To investigate the risk of HPV‐second primary cancers among survivors of HPV‐associated index cancers	113 272 (1397 HPV‐second primary cancers)	Patients who had index oropharyngeal cancers had the highest HPV‐second primary cancer risk (standard incidence ratio, 19.8; 95% CI 18.4–21.4). Women who had index cervical cancers and men who had index anal cancers had the lowest HPV‐second primary cancer risk (standard incidence ratio, 2.4; 95% CI 2.2–2.7)	The HPV‐second primary cancer risk among survivors of HPV‐associated cancers is significant, implying that persistent HPV infection at multiple sites may be associated with HPV‐second primary cancers.
Sabeena et al.^20^	2020	*J Gynecol Oncol*	HPV after radiotherapy and risk of recurrence in cervical cancer	Systematic review and meta‐analysis	To assess the role of HPV DNA testing in early detection of recurrence among cervical cancer survivors after radiotherapy	1055	The overall pooled sensitivity and specificity of HPV DNA testing was 0.84 (95% CI 0.66–0.94) and 0.35 (95% CI 0.20–0.54), respectively. The positive likelihood ratio was 1.3 (95% CI 1.0–1.7) and the negative likelihood ratio was 0.45 (95% CI 0.18–1.10) with an estimated diagnostic odds ratio of 3 (95% CI 1–9)	The screening for HPV DNA testing during follow up facilitates early detection of recurrence after radiotherapy.
Aryasomayajula et al.^21^	2022	*Gynecol Oncol*	HPV testing in cervical cancer surveillance	Retrospective single institution	To assess whether the presence of high‐risk (hr)‐HPV infection after cervical cancer treatment is associated with recurrent disease	262	41 (24%) had at least one positive hr‐HPV test. Recurrent disease was diagnosed in 24 (14%). Of the 24 patients with recurrent disease, 5 (21%) had at least one positive hr‐HPV test during surveillance, versus 36 (24%) of 145 patients without recurrent disease (*P* = 0.67). No recurrences were detected by hr‐HPV testing	Positive hr‐HPV testing in the surveillance setting was not associated with cervical cancer recurrence but did lead to additional studies and procedures
Henderson et al.^22^	2022	*Cancer*	Subsequent malignant neoplasms in the Childhood Cancer Survivor Study: Occurrence of cancer types in which HPV is an established etiologic risk factor	Retrospective (Childhood Cancer Survivor Study)	To estimate the risks for SMN at sites where HPV is an established risk factor and to assess other potential risk factors associated with their development	24 363 (46 developed HPV‐associated‐SMN)	Female survivors were not at increased risk of cervical or vulvar cancers compared with the general population	Childhood cancer survivors are at increased risk for SMN in sites susceptible to HPV‐associated malignancies.
Ou et al.^23^	2023	*Cancer Epidemiol Biomarkers Prev*	HPV‐associated subsequent malignant neoplasms among adolescent and young adult cancer survivors	Retrospective (SEER database)	To identify demographic and clinical risk factors for HPV‐associated SMN among Adolescent and Young Adult cancer survivors	374 408 (1369 HPV‐associated SMN)	Compared with the general population, adolescent and young adult cancer survivors had 70% increased risk for any HPV‐SMN (95% CI 1.61–1.79) and 117% for oropharyngeal‐SMN (95% CI 2.00–2.35); cervical‐SMN risk was generally lower in survivors (SIR, 0.85; 95% CI 0.76–0.95), but Hispanic adolescent and young adult cancer survivors had an 8.4 significant increase in cervical‐SMN (SIR, 1.46; 95% CI 1.01–2.06) Chemotherapy and radiation were associated with any HPV‐SMN among survivors with first HPV‐related cancers, but not associated among survivors whose first cancers were not HPV‐related	HPV‐SMN in adolescent and young adult cancer survivors are driven by oropharyngeal cancers despite temporal declines in oropharyngeal‐SMN. Hispanic survivors are at risk for cervical‐SMN relative to the general population.

Abbreviations: CI, confidence interval; HPV, human papillomavirus; hr., high‐risk; PAYA, pediatric and young adults; SEER, surveillance, epidemiology, and end results; SMN, subsequent malignant neoplasms.

With this background, the available evidence in the literature comes from studies analyzing the use of HPV vaccine after previous HPV exposure and in patients with pre‐invasive disease, showing a reduction of the risk of recurrent cervical intraepithelial neoplasia grade 2 or higher (CIN2^+^) in the order of 60%.^33^, ^34^ In particular, a recent meta‐analysis focused on the role of prophylactic HPV as secondary prevention in patients with HPV‐related disease after primary treatment and concluded that adjuvant HPV vaccination was associated with a reduced risk of CIN recurrence, although there are limited data regarding its role in other HPV‐related diseases.^35^ However, authors of this meta‐analysis acknowledged the heterogeneity between the included studies in terms of inclusion criteria and methodologies, the analysis of both randomized and non‐randomized studies and the potential selection bias, as main limitations of the study.

A very recent study performed a pooled analysis of four large clinical trials worldwide to assess HPV vaccine efficacy and immunogenicity in females with evidence of existing infection and showed that females with existing high‐risk HPV infections at first vaccination still benefit from vaccination in preventing pre‐cancers related to the non‐infected types at baseline. The findings from this study reinforced the importance of vaccinating females irrespective of HPV status in case of a missed pre‐sexual vaccination.^36^

Results from the NOVEL trial, a randomized trial allocating patients with high‐grade CIN to receive HPV vaccination versus no vaccination, are expected in 2025.^37^ It has been shown that vaccination after HSIL diagnosis and treatment is a cost‐effective strategy.^38^ Data are also available on the reduction of recurrent HSIL of the vulva and the anus.^39^

Additionally, an Italian multicentric, randomized, double‐blind, placebo‐controlled, phase III trial (HOPE9 study) is currently ongoing to investigate the efficacy of presurgical HPV vaccination in women treated with conization for CIN2^+^ up to micro‐invasive cervical cancer (FIGO [the International Federation of Gynecology & Obstetrics] stage IA1).^40^

The timing of HPV vaccine administration in cervical cancer is also not established. Focusing on this issue, a recent meta‐analysis assessed the timing of HPV vaccination as adjuvant treatment of HSIL recurrence in women undergoing surgical excision: the authors confirmed that vaccination was beneficial, but were unable to determine whether vaccination, completed or initiated before treatment (conization), would be associated with more favorable results.^41^ A start of vaccination close to surgery seems to be beneficial.^42^

## 4 IS THERE A ROLE FOR PROPHYLACTIC HPV VACCINES AFTER DIAGNOSIS OF CERVICAL CARCINOMA?

To the best of our knowledge, the literature evidence about the use of prophylactic HPV vaccine after a previous diagnosis of invasive cervical cancer is absent. A recent retrospective study analyzed patients who developed lower genital tract dysplasia (including anal, vulvar, and vaginal intraepithelial neoplasia) after having a hysterectomy for CIN2^+^ and FIGO stage IA1–IB1 cervical cancer.^43^ In this study, 15/77 (19.5%) patients were diagnosed with cervical cancer. The authors restricted the analysis to the 18 patients with available HPV data at the time of hysterectomy and postulated the beneficial effect of nonavalent HPV vaccination in 16 patients (16/18; 89%). However, considering that patients with persistent HPV types (i.e. with the same HPV types at the time of hysterectomy and subsequent low genital tract disease) would not benefit from vaccination, the authors estimated the potential protective effect of vaccination to be 67% (12/18 patients), providing a proof‐of‐principle support to design prospective studies testing vaccination in cervical cancer patients. Nevertheless, the results from this study must be taken with caution as the authors did not assess the role of HPV vaccination in preventing or reducing the risk of HPV clearance, re‐infection, or cervical cancer recurrence as no intervention was evaluated. A systematic review and meta‐analysis on the role of HPV vaccination on HPV infection and recurrence of HPV‐related disease after local surgical treatment demonstrated that HPV vaccine might reduce the risk of recurrence of HSIL, in particular when related to HPV16 or HPV18, in women treated with local excision.^44^ However, the quality of studies is low and a higher level of evidence is awaited from the NOVEL trial.^37^ Simply by translating this concept in the setting of invasive cervical cancer, clinicians may advise patients to undergo prophylactic HPV vaccination during or after treatment. This is probably applicable to women with microinvasive cervical cancer treated with conization. It is more difficult to estimate the effect in those treated for higher stage disease or undergoing radical hysterectomy as there are no data; however, we have to keep in mind that they are also at risk for HPV‐related disease in other sites (Table 1). In fact, the vaccination might protect against the development of other HPV‐related diseases, such as vaginal cancer, even if it is always difficult to discriminate whether vaginal carcinoma in a cervical cancer survivor is a primary vaginal cancer or a recurrence of their cervical cancer. It is even more difficult to estimate the benefit of HPV vaccination in those being treated with primary chemo‐radiation, but these patients are more vulnerable after treatment and need the best care possible. Some national programs already reimburse HPV vaccination in women undergoing conization for pre‐invasive disease (Austria, Canada, Italy, Spain) or for invasive cancer (Italy, Brazil, Canada). The safety profile of HPV vaccines might support the use of HPV vaccine in this category of patients.^45^ In Europe, no upper age limit for the recommendation of HPV vaccination exists. Although population‐based data have demonstrated that early vaccination provides most benefit; in individuals, HPV vaccination has been shown to be immunogenic up to the age of 55 years^46^ and to prevent infection/disease at least up to the age of 45 years^47^, ^48^ even though a cost‐effectiveness balance must be taken into account.^6^ On the other hand, other trials such as the Future III and Viviane trials did not show any protection in the intention‐to‐treat analyses.^49^, ^50^

## 5 SHOULD WE OFFER PROPHYLACTIC HPV VACCINES IN PATIENTS WITH OTHER HPV‐RELATED GYNECOLOGIC CANCERS?

Patients with other HPV‐related gynecologic cancers (such as vulvar and vaginal cancers) carry similar risk of HPV infection recurrence or persistence, as in the case of cervical cancer.^51^ Nevertheless, the literature data on the use of HPV vaccination with respect to preventing recurrences in women treated for vulvar and vaginal pre‐invasive lesions are scarce.^15^, ^39^ As for cervical cancer, no literature evidence is available on the benefit of HPV vaccination after diagnosis and treatment of invasive vulvar and vaginal cancers.

Diagnosis and treatment of any invasive HPV‐related gynecologic cancer might represent a moment for any gynecologic oncologist to counsel the patient about the benefits and uncertainty of HPV vaccination.

## 6 CONCLUSIONS

Literature evidence on the use of prophylactic HPV vaccines in patients diagnosed with and treated for HPV‐related gynecologic cancers is absent. Cancer survivors are a vulnerable population with an increased risk for subsequent (HPV‐related) disease and secondary malignancy. They should receive the best protection possible according to evidence‐based information.

It is European Society of Gynecological Oncology (ESGO) prevention committee opinion that there is no evidence to support the use of HPV vaccination in patients with HPV‐related gynecologic cancers and no recommendation can be made. Counseling of these patients should be performed as for any HPV‐related non‐gynecologic cancer (such as anal or oropharyngeal cancer) and non‐cancer patient, suggesting HPV vaccination according to patient's age and prognosis, knowing there is a decrease of efficacy with increasing age. Studies on the use of prophylactic HPV vaccine in patients diagnosed with HPV‐related gynecologic cancers are strongly needed.

## AUTHOR CONTRIBUTIONS

M.G., E.A.J., and N.B. contributed to conceptualization, methodology, and software; E.A.J. and N.B. contributed to data curation and wrote the original draft; I.Z. performed the visualization and investigation; M.G., E.A.J. supervised the study; and M.K., R.D.V., Z.R., N.T., I.T., and C.T. contributed to the data curation, software, and validation. All authors contributed to writing—reviewing and editing.

## CONFLICT OF INTEREST STATEMENT

MG is supported by Horizon 2020 Framework Programme for Research and Innovation of the European Commission, through the RISCC Network. All other authors have no conflicts of interest.

## Data Availability

Data sharing is not applicable to this article as no new data were created or analyzed in this study.
